# Cross-Protection between West Nile Virus and Emerging Flaviviruses in Wild Birds

**DOI:** 10.4269/ajtmh.24-0363

**Published:** 2024-12-31

**Authors:** Angela M. Bosco-Lauth, Kris Kooi, Seth A. Hawks, Nisha K. Duggal

**Affiliations:** ^1^ Department of Biomedical Sciences, Colorado State University, Fort Collins, Colorado;; ^2^ Wildlife Services, Animal and Plant Health Inspection Service, U.S. Department of Agriculture, Golden, Colorado;; ^3^ Department of Biomedical Sciences and Pathobiology, Virginia Polytechnic Institute and State University, Blacksburg, Virginia

## Abstract

West Nile virus (WNV), St. Louis encephalitis virus (SLEV), and Usutu virus (USUV) are zoonotic flaviviruses that cause neuroinvasive disease in humans and are maintained in overlapping avian-mosquito transmission cycles. West Nile virus and SLEV cocirculate in the United States, and WNV and USUV cocirculate in Europe. Cross-reactivity of immune responses against closely related flaviviruses is well documented. In birds, prior infection with WNV provides strong protection against SLEV genotype II and V infection, which may explain the decrease in SLEV circulation in the United States after WNV emergence in 1999. However, in 2015, a new SLEV genotype (III) emerged in the United States, suggesting that WNV immunity in birds may not provide cross-protection against this SLEV genotype. Here, we tested whether prior WNV infection protects birds against infection with SLEV genotype III, as well as USUV. First, we established a house sparrow (*Passer domesticus*) model of infection for SLEV genotype III. We then inoculated house sparrows with WNV and, 4 weeks later, challenged WNV-immune birds with SLEV genotype III or USUV. All birds were completely protected against secondary challenge, with no viremia detected. Low levels of cross-neutralizing antibodies against SLEV and USUV were found in the blood prior to secondary challenge. However, two naturally WNV-exposed house sparrows did develop SLEV genotype III and USUV viremia after inoculation. These results indicate that experimental WNV infection may protect birds against infection with SLEV genotype III and USUV; however, additional studies to investigate the role of avian immunity in flavivirus emergence are necessary.

## INTRODUCTION

West Nile virus (WNV), St. Louis encephalitis virus (SLEV), and Usutu virus (USUV) are antigenically related flaviviruses within the Japanese encephalitis serogroup. West Nile virus, SLEV, and USUV are maintained in avian-mosquito transmission cycles, with overlapping species of passerine birds as reservoirs and *Culex* mosquitoes as vectors. There are geographic areas of overlap among these viruses, including North and South America for WNV and SLEV and Europe and Africa for WNV and USUV. Furthermore, immune responses against WNV, SLEV, and USUV cross-react, with cross-protection observed in mammalian models of infection.^1^^,^^2^

Because of the overlapping avian reservoirs and the cross-reactive immune responses between these viruses, there is a possibility of heterologous virus displacement within birds. In wild birds, prior infection with WNV is protective against secondary WNV infection, as well as secondary infection with SLEV genotypes II and V.^3^^,^^4^ SLEV genotypes I, II, and V have been historically detected in the United States, although SLEV circulation decreased in the United States after the introduction of WNV in 1999. The decrease in SLEV circulation after the introduction of WNV may be due to the cross-reactive immune responses between WNV and SLEV in birds.^5^ However, an outbreak of SLEV in Arizona in 2015 led to the identification of a newly introduced SLEV genotype III in the United States, which is now cocirculating with WNV.^6^^,^^7^ Additionally, WNV cocirculates with USUV in Europe, with individual birds occasionally testing positive for both WNV and USUV by polymerase chain reaction (PCR) and by serology.^8^^,^^9^ This ongoing cocirculation suggests that SLEV genotype III and USUV may not be sensitive to cross-reactive WNV immune responses in birds, and thus, WNV circulation may not be sufficient to prevent heterologous flavivirus emergence.

House sparrows are a major reservoir species for WNV and SLEV transmission in the United States. Not only are house sparrows frequently infected with WNV and SLEV in the United Statees^10^^–^^14^ and with USUV in Europe,^15^ but experimentally infected house sparrows generate viremia sufficient to transmit WNV, SLEV (genotypes II and V), and USUV to biting *Culex* mosquitoes, demonstrating that they are competent reservoirs.^16^^–^^18^ In addition, they are ubiquitous across North America and are a frequent bloodmeal source for *Culex* mosquitoes.^19^^–^^21^

To assess the impact of prior WNV exposure on the newly introduced SLEV genotype III in wild birds, we first developed a house sparrow model of SLEV genotype III infection. We then infected house sparrows with WNV, followed by SLEV genotype III or USUV. Low levels of cross-neutralizing antibodies against SLEV genotype III and USUV were generated by primary WNV infection. No secondary SLEV or USUV viremia was detected in birds experimentally infected with WNV. However, SLEV and USUV viremia was detected in two birds with natural WNV exposure.

## MATERIALS AND METHODS

### Viruses.

The following viruses were used in these studies: WNV strain M19433 (Texas 2007)^22^ passage 4, SLEV genotype III strain RT121B (Arizona 2015)^23^ passage 4, USUV strain TMNetherlands (Netherlands 2016)^24^ passage 5, and USUV strain UG09615 (Uganda 2012)^25^ passage 3. Usutu virus stocks were previously sequenced by our laboratory.^1^^,^^26^ West Nile virus and SLEV stocks were sequenced by amplifying the genome in overlapping PCR products, followed by Sanger sequencing, and confirmed to be identical to published sequences.

### Cells.

Vero cells were cultured in Dulbecco’s modified Eagle medium supplemented with 5% fetal bovine serum (FBS) and 1% penicillin-streptomycin. Cells were maintained at 37°C with 5% CO_2_.

### Virus quantification.

Samples were tested for WNV, SLEV, or USUV titers by standard Vero cell plaque assay as previously described.^27^ Briefly, six-well plates with confluent monolayers of cells were inoculated with 100 µL of serial 10-fold dilutions of samples, incubated for 1 hour at 37°C, and overlaid with 0.5% agarose in minimal essential medium containing 2% FBS and antibiotics/antifungal agents. A second overlay containing neutral red indicator dye was added 48 hours later for WNV, 72 hours later for USUV, and 6 days later for SLEV. Plaques were counted on days 3, 4, and 7 for WNV, USUV, and SLEV, respectively. The limit of detection was 100 plaque-forming units (PFU)/mL (2 log_10_ PFU/mL). Samples with no plaques were assigned half the limit of detection, or 50 PFU/mL (1.7 log_10_ PFU/mL).

### Ninety percent plaque reduction neutralization test (PRNT_90_) assays.

Serum samples were heat inactivated at 56°C for 30 minutes and incubated with approximately 100 PFU of WNV, SLEV, or USUV for 1 hour, followed by a standard Vero cell plaque assay.^27^ For WNV neutralization screening prior to challenge, a 1:10 dilution was performed, and samples were considered seronegative if there was less than 50% reduction in plaques. For further titration of seropositive samples, an additional five serial 2-fold dilutions were performed (1:20 through 1:640). For neutralization tests after challenge, a 1:40 dilution followed by five serial 2-fold dilutions was used (1:40 through 1:1280). For titrations, neutralization was defined by 90% reduction in plaques compared with a negative control. The limit of detection for neutralization screening prior to challenge was 1:10, and the limit of detection for neutralization tests after challenge was 1:40. Samples that did not reach 90% neutralization were assigned half the limit of detection, or 1:5 for neutralization tests prior to challenge and 1:20 for neutralization tests after challenge.

### House sparrow studies.

Forty-eight house sparrows were mist-netted in Larimer and Weld counties in Colorado in 2022. A blood sample was collected prior to inoculation to test for previous WNV exposure by PRNT. Seven birds (15%) were seropositive for WNV. A subset of seronegative house sparrows was inoculated subcutaneously with 1,500 PFU of SLEV strain RT121B (*n* = 13) or USUV strain Netherlands (*n* = 5), and blood samples were collected on day post-inoculation (dpi) 1, 2, 3, 5, and 7 and tested for viral titer. Then, another group of seronegative house sparrows was inoculated subcutaneously with 1,500 PFU of WNV (*n* = 23), and blood samples were collected on dpi 1, 3, and 5 and tested for viral titer. A blood sample was collected on dpi 25 and tested for neutralizing antibodies by PRNT. Three birds did not survive to the secondary challenge and were excluded from analyses. Twenty-eight days after primary WNV challenge, house sparrows were inoculated subcutaneously with 1,500 PFU of SLEV strain RT121B (*n* = 6), USUV strain Netherlands 2016 (*n* = 7), or USUV strain Uganda 2012 (*n* = 7). Blood samples were collected after secondary challenge on dpi 1, 2, 3, 5, and 7 and tested for viral titer. Naturally seropositive birds were similarly inoculated with 1,500 PFU of WNV (*n* = 5), SLEV strain RT121B (*n* = 1), or USUV strain Netherlands (*n* = 1), and blood samples were collected and tested for viral titer.

## STATISTICAL ANALYSES

Data were analyzed using GraphPad Prism 10, including descriptive statistics and linear regression.

## RESULTS

### House sparrow model of SLEV genotype III infection.

**Figure 1.**
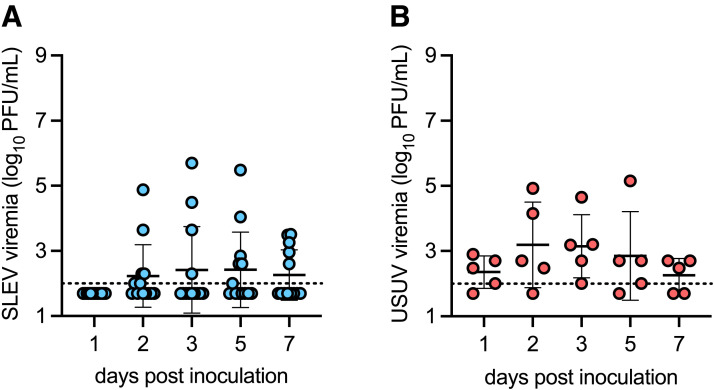
St. Louis encephalitis virus (SLEV) genotype III and Usutu virus (USUV) infection in West Nile virus (WNV)-naïve house sparrows. (**A**) Viremia in house sparrows inoculated with SLEV strain Arizona 2015 (*n* = 13). (**B**) Viremia in house sparrows inoculated with USUV strain Netherlands 2016 (*n* = 5). Circles represent individual birds; lines represent means; error bars represent standard deviations. The dashed line represents the limit of detection.

House sparrows were collected in Colorado and tested for WNV-neutralizing antibodies by PRNT_90_. One group of seronegative house sparrows was inoculated with SLEV strain Arizona 2015, which is a genotype III strain. Ninety-two percent (12/13) of SLEV-inoculated birds developed detectable viremia, with a mean peak viremia of 2.4 log_10_ PFU/mL on dpi 3 and 5 (Figure 1A). This is similar to the previously reported mean peak viremia in house sparrows for SLEV strain Kern217, which is a genotype II strain, and is sufficient to transmit SLEV to a biting *Culex* mosquito.^16^ As a positive control, another group of seronegative house sparrows was inoculated with USUV strain Netherlands 2016, a model we previously established using house sparrows collected in Virginia.^18^ All USUV-inoculated birds developed detectable viremia, with a peak mean viremia of 3.0 log_10_ PFU/mL on dpi 2 (Figure 1B). This result is consistent with our previously observed peak mean viremia and is also sufficient to transmit USUV to a biting *Culex* mosquito.^18^ Together, these data indicate that house sparrows are an appropriate model for SLEV genotype III and USUV infection in birds.

### Cross-protection against SLEV genotype III and USUV in house sparrows after experimental WNV infection.

**Figure 2.**
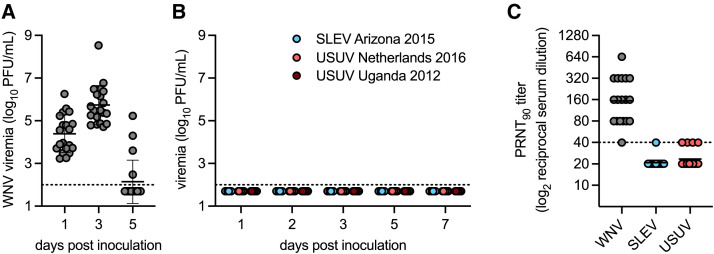
St. Louis encephalitis virus (SLEV) genotype III and Usutu virus (USUV) infection in experimentally West Nile virus (WNV)-exposed house sparrows. (**A**) Viremia in house sparrows inoculated with WNV strain Texas 2007 (*n* = 20). (**B**) Viremia in experimentally WNV-exposed house sparrows inoculated with SLEV strain Arizona 2015 (*n* = 6), USUV strain Netherlands 2016 (*n* = 7), or USUV strain Uganda 2012 (*n* = 7). (**C**) Ninety percent plaque reduction neutralization titers (PRNT_90_ titers) in house sparrows after inoculation with WNV strain Texas 2007 (*n* = 18) against WNV, SLEV, and USUV. Circles represent individual birds; lines represent means; error bars represent the standard deviations. The dashed line represents the limit of detection.

To determine whether prior WNV exposure protects birds against SLEV genotype III and USUV infection, seronegative house sparrows were inoculated with WNV strain Texas 2007. All house sparrows developed robust WNV viremia, which peaked on dpi 3 with a mean titer of 5.7 log_10_ PFU/mL (Figure 2A). Twenty-eight days after primary infection, house sparrows were inoculated with SLEV strain Arizona 2015, USUV strain Netherlands 2016, or USUV strain Uganda 2012. All birds were protected against secondary infection, with no detectable SLEV or USUV viremia in any sample (Figure 2B), indicating strong cross-protection against SLEV and USUV.

Next, we tested whether WNV infection generates neutralizing antibody responses that cross-neutralize SLEV genotype III or USUV. Serum samples from the experimentally WNV-infected house sparrows were collected 25 days after WNV inoculation, prior to secondary challenge with SLEV or USUV. These serum samples were subjected to PRNT_90_ against WNV strain Texas 2007, SLEV strain Arizona 2015, and USUV strain Netherlands 2016. All WNV-inoculated house sparrows developed a neutralizing antibody response against WNV, with a geometric mean titer of 154 (Figure 2C). Six percent (1/18) of the WNV-inoculated house sparrows had detectable neutralizing antibody responses against SLEV, and 22% (4/18) had detectable neutralizing antibody responses against USUV (Figure 2C). This suggests that low levels of cross-neutralizing antibodies are generated after WNV infection, which may contribute to the cross-protection against SLEV and USUV infection.

### Cross-protection against SLEV genotype III and USUV in house sparrows after natural WNV infection.

**Figure 3.**
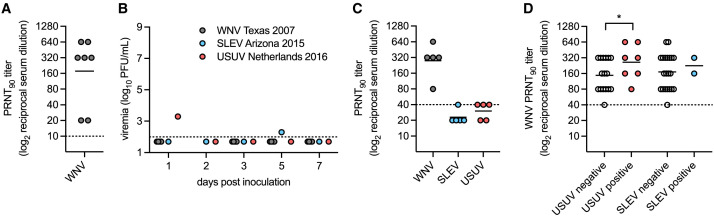
St. Louis encephalitis virus (SLEV) genotype III and Usutu virus (USUV) infection in naturally West Nile virus (WNV)-exposed house sparrows. (**A**) WNV ninety percent plaque reduction neutralization titers (PRNT_90_ titers) in naturally seropositive house sparrows prior to experimental infection (*n* = 7). (**B**) Viremia in naturally WNV-exposed house sparrows inoculated with WNV strain Texas 2007 (*n* = 5), SLEV strain Arizona 2015 (*n* = 1), or USUV strain Netherlands 2016 (*n* = 1). (**C**) PRNT_90_ titers in house sparrows naturally WNV exposed, followed by inoculation with WNV strain Texas 2007 (*n* = 5) against WNV, SLEV, and USUV. (**D**) WNV PRNT_90_ titers in samples with or without cross-reactivity to USUV or SLEV. *P <0.05. Circles represent individual birds, and lines represent the geometric means. The dashed line represents the limit of detection.

Seven naturally WNV-seropositive birds were also inoculated with WNV Texas 2007, SLEV Arizona 2015, or USUV Netherlands 2016. The geometric mean PRNT_90_ titer against WNV for the naturally WNV-seropositive birds was 177 (Figure 3A), which was not significantly different from the mean PRNT_90_ titer of 154 for the experimentally WNV-seropositive birds against WNV (Figure 2C). Interestingly, while 0% (0/5) of the naturally WNV-exposed house sparrows developed WNV viremia, 100% (2/2) of the naturally WNV-immune house sparrows did develop detectable SLEV or USUV viremia (Figure 3B). For these two birds, the PRNT_90_ titers against WNV were 20 and 640. This suggests that WNV immunity against heterologous viruses may differ from immunity against WNV.

To determine if multiple WNV exposures increased cross-neutralizing responses, we also tested sera from the five house sparrows that were both naturally WNV exposed and WNV inoculated for neutralizing responses. Serum samples was collected 25 days after WNV inoculation. These WNV-boosted house sparrows had a slightly higher mean PRNT_90_ titer against WNV, of 279 (Figure 3C), than house sparrows with a single WNV exposure, which had a WNV PRNT_90_ titer of 154 (Figure 2C). Additionally, more of the WNV-boosted house sparrows had detectable neutralizing antibody responses against SLEV (20%, 1/5) and USUV (60%, 3/5) (Figure 3C) than did house sparrows with a single WNV exposure (6% and 22%, respectively) (Figure 2C). To determine whether there was a relationship between the magnitude of WNV PRNT_90_ titer and cross-reactivity with SLEV or USUV, WNV PRNT_90_ titers were compared between samples with and without cross-reactivity to SLEV or USUV. For samples with cross-reactivity to USUV, there was a significantly higher WNV PRNT_90_ titer than with samples with no cross-reactivity to USUV (Figure 3D, *P* <0.05). There was no difference in WNV PRNT_90_ titer between samples with and without cross-reactivity to SLEV (Figure 3D), which could be due to the low numbers of samples that neutralized SLEV. These results suggest that the magnitude of neutralizing immune responses against WNV impacts the level of cross-neutralizing responses against heterologous viruses.

## DISCUSSION

In this study, we established that house sparrows are competent for SLEV genotype III, as they developed sufficient viremia to transmit to a biting *Culex* mosquito (Figure 1). Experimental WNV infection protected house sparrows against secondary infection with SLEV genotype III and USUV and induced weakly cross-neutralizing responses against SLEV genotype III and USUV (Figure 2). However, two house sparrows with natural WNV infection developed detectable SLEV genotype III and USUV viremia (Figure 3), suggesting that further studies on the duration of cross-protective immunity are needed.

The level of SLEV genotype III viremia that we detected in inoculated house sparrows was similar to viremia generated by a SLEV genotype II strain in wild birds, although the viremia was lower than with a SLEV genotype V strain.^4^^,^^16^ In addition, SLEV genotype III viremia was lower than USUV or WNV viremia in wild birds, which is consistent with other reports.^3^^,^^4^^,^^16^^,^^18^ Increasing doses of SLEV in susceptible birds have not been shown to increase viremia.^28^ Some genetic determinants of flavivirus replication in birds have been mapped in WNV and SLEV and are located primarily in the nonstructural genes.^4^^,^^29^^,^^30^ Differences in viremia generated by USUV genotypes have not been reported in birds, although USUV strains can differ significantly in viremia and pathogenesis in mammalian models.^26^

Our results with experimental WNV infection in house sparrows indicate that WNV immune responses in recently infected birds are capable of preventing SLEV genotype III and USUV infection. While we tested WNV lineage 1 in our study, WNV lineages 1 and 2 circulate in Europe. Thus, the cross-protection between WNV lineage 2 and USUV in birds would be important to evaluate in the future. Our results are in line with previous studies demonstrating that experimental infection of house finches and house sparrows with WNV provided birds with complete protection against SLEV genotype II and V infection, with no viremia detected.^3^^,^^4^ Interestingly, in the reciprocal experiment, infection of house finches and house sparrows with SLEV genotype II or V decreased subsequent WNV viremia but did not prevent infection.^3^^,^^4^ This suggested that WNV may be able to exclude SLEV in wild bird populations, but SLEV infection may not provide the same level of cross-protection. Similarly, USUV infection has been shown to provide only partial protection against secondary WNV infection in birds.^31^

The duration of WNV immunity in house sparrows has been studied experimentally. Neutralizing antibody responses against WNV and complete protection against WNV infection were maintained for 3 years in house sparrows after experimental WNV inoculation.^32^ Whether natural WNV infection leads to a similar duration in neutralizing antibody responses and protection is not known. For SLEV, the duration of protective immune responses is more variable, and exposure to SLEV can be protective against SLEV even in the absence of detectable neutralizing antibody responses,^13^^,^^33^^,^^34^ raising the possibility that protection is mediated by other mechanisms, such as cellular immunity. In mammals, CD8^+^ T cells have been demonstrated to be critical for protection against WNV disease.^35^^–^^38^

Although all experimentally WNV-infected house sparrows were protected against SLEV and USUV infection in our study, not all house sparrows developed detectable neutralizing antibodies. This suggests that either low levels (below our limit of detection) of cross-neutralizing antibodies are required for protection or other adaptive responses such as cellular immunity might be important for protection. In mammals, passive transfer studies have demonstrated that WNV-neutralizing antibodies are protective against WNV infection,^39^ and WNV-neutralizing antibodies can cross-protect against heterologous flavivirus infection.^40^ In contrast, in birds, maternally transferred neutralizing antibodies against WNV are short-lived and not protective in house sparrow chicks.^41^ Ultimately, however, the reemergence of SLEV genotype III in the United States after WNV was introduced demonstrates that WNV immunity in birds is not sufficient to prevent heterologous flavivirus emergence. In the recent outbreak of SLEV in the United States, SLEV and WNV cocirculated in the same geographic area within overlapping mosquito populations and caused outbreaks of disease in humans concurrently.^7^ This could be due to waning levels of cross-reactive WNV immunity in avian populations, which has not been studied.

However, WNV seroprevalence in wild bird populations, while variable from year to year, appears to be maintained in the United States,^42^^,^^43^ although the magnitude or breadth of the natural WNV immune response in birds has not been investigated over time. Our results suggest that WNV immunity provides cross-protection against SLEV genotype III and USUV infection in birds for at least 1 month, although further studies to assess the duration of cross-protective immune responses are needed.

## References

[1] Salgado R Hawks SA Frere F Vazquez A Huang CY Duggal NK, 2021. West Nile virus vaccination protects against Usutu virus disease in mice. Viruses 13: 2352. 34960621 10.3390/v13122352 PMC8704473

[2] Blazquez AB Escribano-Romero E Martin-Acebes MA Petrovic T Saiz JC, 2015. Limited susceptibility of mice to Usutu virus (USUV) infection and induction of flavivirus cross-protective immunity. Virology 482: 67–71. 25827530 10.1016/j.virol.2015.03.020

[3] Fang Y Reisen WK, 2006. Previous infection with West Nile or St. Louis encephalitis viruses provides cross protection during reinfection in house finches. Am J Trop Med Hyg 75: 480–485. 16968925

[4] Maharaj PD Bosco-Lauth AM Langevin SA Anishchenko M Bowen RA Reisen WK Brault AC, 2018. West Nile and St. Louis encephalitis viral genetic determinants of avian host competence. PLoS Negl Trop Dis 12: e0006302. 29447156 10.1371/journal.pntd.0006302 PMC5831645

[5] Reisen WK Lothrop HD Wheeler SS Kennsington M Gutierrez A Fang Y Garcia S Lothrop B, 2008. Persistent West Nile virus transmission and the apparent displacement St. Louis encephalitis virus in southeastern California, 2003–2006. J Med Entomol 45: 494–508. 18533445 10.1603/0022-2585(2008)45[494:pwnvta]2.0.co;2 PMC2435167

[6] White GS Symmes K Sun P Fang Y Garcia S Steiner C Smith K Reisen WK Coffey LL, 2016. Reemergence of St. Louis encephalitis virus, California, 2015. Emerg Infect Dis 22: 2185–2188. 27869600 10.3201/eid2212.160805 PMC5189155

[7] Venkat H , 2015. Concurrent outbreaks of St. Louis encephalitis virus and West Nile virus disease—Arizona, 2015. MMWR Morb Mortal Wkly Rep 64: 1349–1350. 26656306 10.15585/mmwr.mm6448a5

[8] Buckley A Dawson A Moss SR Hinsley SA Bellamy PE Gould EA, 2003. Serological evidence of West Nile virus, Usutu virus and Sindbis virus infection of birds in the UK. J Gen Virol 84: 2807–2817. 13679615 10.1099/vir.0.19341-0

[9] Tamba M Bonilauri P Bellini R Calzolari M Albieri A Sambri V Dottori M Angelini P, 2011. Detection of Usutu virus within a West Nile virus surveillance program in Northern Italy. Vector Borne Zoonotic Dis 11: 551–557. 20849275 10.1089/vbz.2010.0055

[10] Gibbs SE Allison AB Yabsley MJ Mead DG Wilcox BR Stallknecht DE, 2006. West Nile virus antibodies in avian species of Georgia, USA: 2000–2004. Vector Borne Zoonotic Dis 6: 57–72. 16584328 10.1089/vbz.2006.6.57

[11] Beveroth TA Ward MP Lampman RL Ringia AM Novak RJ, 2006. Changes in seroprevalence of West Nile virus across Illinois in free-ranging birds from 2001 through 2004. Am J Trop Med Hyg 74: 174–179. 16407365

[12] Kwan JL Kluh S Reisen WK, 2012. Antecedent avian immunity limits tangential transmission of West Nile virus to humans. PLoS One 7: e34127. 22457819 10.1371/journal.pone.0034127 PMC3311586

[13] McLean RG Mullenix J Kerschner J Hamm J, 1983. The house sparrow (*Passer domesticus*) as a sentinel for St. Louis encephalitis virus. Am J Trop Med Hyg 32: 1120–1129. 6312819 10.4269/ajtmh.1983.32.1120

[14] Gruwell JA Fogarty CL Bennett SG Challet GL Vanderpool KS Jozan M Webb JP Jr. 2000. Role of peridomestic birds in the transmission of St. Louis encephalitis virus in southern California. J Wildl Dis 36: 13–34. 10682741 10.7589/0090-3558-36.1.13

[15] Steinmetz HW Bakonyi T Weissenbock H Hatt JM Eulenberger U Robert N Hoop R Nowotny N, 2011. Emergence and establishment of Usutu virus infection in wild and captive avian species in and around Zurich, Switzerland—Genomic and pathologic comparison to other central European outbreaks. Vet Microbiol 148: 207–212. 20980109 10.1016/j.vetmic.2010.09.018

[16] Reisen WK Fang Y Martinez VM, 2005. Avian host and mosquito (Diptera: Culicidae) vector competence determine the efficiency of West Nile and St. Louis encephalitis virus transmission. J Med Entomol 42: 367–375. 15962789 10.1093/jmedent/42.3.367

[17] Komar N Langevin S Hinten S Nemeth N Edwards E Hettler D Davis B Bowen R Bunning M, 2003. Experimental infection of North American birds with the New York 1999 strain of West Nile virus. Emerg Infect Dis 9: 311–322. 12643825 10.3201/eid0903.020628 PMC2958552

[18] Kuchinsky SC Marano J Hawks SA Loessberg E Honaker CF Siegel PB Lahondère C LeRoith T Weger-Lucarelli J Duggal NK, 2022. North American house sparrows are competent for Usutu virus transmission. mSphere 7: e0029522. 36317895 10.1128/msphere.00295-22 PMC9769741

[19] Hamer GL Kitron UD Goldberg TL Brawn JD Loss SR Ruiz MO Hayes DB Walker ED, 2009. Host selection by *Culex pipiens* mosquitoes and West Nile virus amplification. Am J Trop Med Hyg 80: 268–278. 19190226

[20] Thiemann TC Wheeler SS Barker CM Reisen WK, 2011. Mosquito host selection varies seasonally with host availability and mosquito density. PLoS Negl Trop Dis 5: e1452. 22206038 10.1371/journal.pntd.0001452 PMC3243726

[21] Komar N Panella NA Young GR Brault AC Levy CE, 2013. Avian hosts of West Nile virus in Arizona. Am J Trop Med Hyg 89: 474–481. 23857022 10.4269/ajtmh.13-0061 PMC3771284

[22] McMullen AR May FJ Li L Guzman H Bueno R Jr. Dennett JA Tesh RB Barrett AD, 2011. Evolution of new genotype of West Nile virus in North America. Emerg Infect Dis 17: 785–793. 21529385 10.3201/eid1705.101707 PMC3321787

[23] Swetnam DM , 2020. Movement of St. Louis encephalitis virus in the Western United States, 2014–2018. PLoS Negl Trop Dis 14: e0008343. 32520944 10.1371/journal.pntd.0008343 PMC7307790

[24] Rijks JM , 2016. Widespread Usutu virus outbreak in birds in the Netherlands, 2016. Euro Surveill 21: 30391. 27918257 10.2807/1560-7917.ES.2016.21.45.30391 PMC5144937

[25] Mossel EC Crabtree MB Mutebi JP Lutwama JJ Borland EM Powers AM Miller BR, 2017. Arboviruses isolated from mosquitoes collected in Uganda, 2008–2012. J Med Entomol 54: 1403–1409. 28874015 10.1093/jme/tjx120 PMC5968633

[26] Kuchinsky SC Hawks SA Mossel EC Coutermarsh-Ott S Duggal NK, 2020. Differential pathogenesis of Usutu virus isolates in mice. PLoS Negl Trop Dis 14: e0008765. 33044987 10.1371/journal.pntd.0008765 PMC7580916

[27] Bunning ML , 2002. Experimental infection of horses with West Nile virus. Emerg Infect Dis 8: 380–386. 11971771 10.3201/eid0804.010239 PMC3393377

[28] Robert McLean TS, 1979. Avian hosts of St. Louis encephalitis virus. Bird Control Seminars Proceedings 20: 143–155.

[29] Brault AC Huang CY Langevin SA Kinney RM Bowen RA Ramey WN Panella NA Holmes EC Powers AM Miller BR, 2007. A single positively selected West Nile viral mutation confers increased virogenesis in American crows. Nat Genet 39: 1162–1166. 17694056 10.1038/ng2097 PMC2291521

[30] Dietrich EA Langevin SA Huang CY Maharaj PD Delorey MJ Bowen RA Kinney RM Brault AC, 2016. West Nile virus temperature sensitivity and avian virulence are modulated by NS1-2B polymorphisms. PLoS Negl Trop Dis 10: e0004938. 27548738 10.1371/journal.pntd.0004938 PMC4993437

[31] Reemtsma H Holicki CM Fast C Bergmann F Groschup MH Ziegler U, 2023. A prior Usutu virus infection can protect geese from severe West Nile disease. Pathogens 12: 959. 37513806 10.3390/pathogens12070959 PMC10386565

[32] Nemeth NM Oesterle PT Bowen RA, 2009. Humoral immunity to West Nile virus is long-lasting and protective in the house sparrow (*Passer domesticus*). Am J Trop Med Hyg 80: 864–869. 19407139 PMC2693945

[33] Reisen WK Kramer LD Chiles RE Green EG Martinez VM, 2001. Encephalitis virus persistence in California birds: Preliminary studies with house finches. J Med Entomol 38: 393–399. 11372964 10.1603/0022-2585-38.3.393

[34] Reisen WK Chiles RE Green EN Fang Y Mahmood F, 2003. Previous infection protects house finches from re-infection with St. Louis encephalitis virus. J Med Entomol 40: 300–305. 12943108 10.1603/0022-2585-40.3.300

[35] Sitati EM Diamond MS, 2006. CD4^+^ T-cell responses are required for clearance of West Nile virus from the central nervous system. J Virol 80: 12060–12069. 17035323 10.1128/JVI.01650-06 PMC1676257

[36] Purtha WE Myers N Mitaksov V Sitati E Connolly J Fremont DH Hansen TH Diamond MS, 2007. Antigen-specific cytotoxic T lymphocytes protect against lethal West Nile virus encephalitis. Eur J Immunol 37: 1845–1854. 17559174 10.1002/eji.200737192

[37] Shrestha B Diamond MS, 2004. Role of CD8^+^ T cells in control of West Nile virus infection. J Virol 78: 8312–8321. 15254203 10.1128/JVI.78.15.8312-8321.2004 PMC446114

[38] Parsons R , 2008. The memory T cell response to West Nile virus in symptomatic humans following natural infection is not influenced by age and is dominated by a restricted set of CD8^+^ T cell epitopes. J Immunol 181: 1563–1572. 18606712 10.4049/jimmunol.181.2.1563

[39] Engle MJ Diamond MS, 2003. Antibody prophylaxis and therapy against West Nile virus infection in wild-type and immunodeficient mice. J Virol 77: 12941–12949. 14645550 10.1128/JVI.77.24.12941-12949.2003 PMC296058

[40] Yang MJ Luo HR Fan ZY Feng YX Wei N Zhu BB Ye J Cao SB Si YH, 2023. Development and evaluation of neutralizing antibodies for cross-protection against West Nile virus and Japanese encephalitis virus. Infect Med (Beijing) 2: 212–223. 38073882 10.1016/j.imj.2023.09.001 PMC10699678

[41] Nemeth NM Oesterle PT Bowen RA, 2008. Passive immunity to West Nile virus provides limited protection in a common passerine species. Am J Trop Med Hyg 79: 283–290. 18689637

[42] McMillan JR , 2023. Multi-year comparison of community- and species-level West Nile virus antibody prevalence in birds from Atlanta, Georgia and Chicago, Illinois, 2005–2016. Am J Trop Med Hyg 108: 366–376. 36572005 10.4269/ajtmh.21-1086 PMC9896344

[43] Reisen WK Wheeler SS, 2016. Surveys for antibodies against mosquitoborne encephalitis viruses in California birds, 1996–2013. Vector Borne Zoonotic Dis 16: 264–282. 26974395 10.1089/vbz.2015.1888 PMC4800269

